# Epigenetic Silencing of *LRP2* Is Associated with Dedifferentiation and Poor Survival in Multiple Solid Tumor Types

**DOI:** 10.3390/cancers15061830

**Published:** 2023-03-17

**Authors:** Martin Q. Rasmussen, Gitte Tindbæk, Morten Muhlig Nielsen, Camilla Merrild, Torben Steiniche, Jakob Skou Pedersen, Søren K. Moestrup, Søren E. Degn, Mette Madsen

**Affiliations:** 1 Department of Biomedicine, Aarhus University, 8000 Aarhus, Denmark; 2 Department of Clinical Biochemistry, Horsens Regional Hospital, 8700 Horsens, Denmark; 3 Department of Molecular Medicine (MOMA), Aarhus University Hospital, 8200 Aarhus, Denmark; 4 Department of Clinical Medicine, Aarhus University, 8200 Aarhus, Denmark; 5 Department of Pathology, Aarhus University Hospital, 8200 Aarhus, Denmark; 6 Bioinformatics Research Center (BiRC), Aarhus University, 8000 Aarhus, Denmark; 7 Department of Cancer and Inflammation Research, University of Southern Denmark, 5230 Odense, Denmark

**Keywords:** LRP2, megalin, epigenetics, methylation, tumor dedifferentiation, cancer biomarker

## Abstract

**Simple Summary:**

Epithelial tissues are the most common sites for the development of cancer. Loss of epithelial cell characteristics and dedifferentiation are hallmarks of cancer. A specialized and complex function in epithelial cells is receptor-mediated endocytosis. LRP2 (megalin) is the largest known endocytic membrane receptor of absorptive epithelia and mediates uptake of numerous ligands. However, its role and regulation in cancer has not been delineated. Therefore, we examined *LRP2* expression across 33 cancer types in The Cancer Genome Atlas. As expected, we found the highest *LRP2* expression in cancers arising from LRP2-expressing epithelia. However, in a subset of these tumors, we observed epigenetic silencing of *LRP2*. Interestingly, low *LRP2* expression was associated with tumor cell dedifferentiation and poorer patient outcome, suggesting LRP2 is a potential cancer biomarker. Based on this, we warrant further studies on the functional role of LRP2 in tumors of epithelial origin and the implications of LRP2 downregulation.

**Abstract:**

More than 80% of human cancers originate in epithelial tissues. Loss of epithelial cell characteristics are hallmarks of tumor development. Receptor-mediated endocytosis is a key function of absorptive epithelial cells with importance for cellular and organismal homeostasis. LRP2 (megalin) is the largest known endocytic membrane receptor and is essential for endocytosis of various ligands in specialized epithelia, including the proximal tubules of the kidney, the thyroid gland, and breast glandular epithelium. However, the role and regulation of LRP2 in cancers that arise from these tissues has not been delineated. Here, we examined the expression of *LRP2* across 33 cancer types in The Cancer Genome Atlas. As expected, the highest levels of *LRP2* were found in cancer types that arise from LRP2-expressing absorptive epithelial cells. However, in a subset of tumors from these cancer types, we observed epigenetic silencing of *LRP2. LRP2* expression showed a strong inverse correlation to methylation of a specific CpG site (cg02361027) in the first intron of the *LRP2* gene. Interestingly, low expression of *LRP2* was associated with poor patient outcome in clear cell renal cell carcinoma, papillary renal cell carcinoma, mesothelioma, papillary thyroid carcinoma, and invasive breast carcinoma. Furthermore, loss of *LRP2* expression was associated with dedifferentiated histological and molecular subtypes of these cancers. These observations now motivate further studies on the functional role of LRP2 in tumors of epithelial origin and the potential use of LRP2 as a cancer biomarker.

## 1. Introduction

Epithelial tissues are the most common sites for the development of cancer. Loss of epithelial cell characteristics, such as cell–cell adhesion, cellular polarization, and cell anchorage are hallmarks of tumor development and occur as part of a coordinated epigenetic process named epithelial-to-mesenchymal transition (EMT). EMT is driven by the activation of EMT transcription factors causing global repression of epithelial gene expression [1,2]. Cancer cells can exist along a spectrum of EMT cell states, and this variation contributes to tumor heterogeneity [3]. The acquisition of a more mesenchymal phenotype often results in increased cell migration and invasion; therefore, it has a major impact on tumor development, tumor progression, and metastasis [4]. 

Receptor-mediated endocytosis is a key function of epithelial cells [5]. During receptor-mediated endocytosis, extracellular ligands are internalized following binding to endocytic transmembrane receptors. Upon internalization and detachment of endocytic vesicles with their cargo, receptors and ligands traffic intracellularly in endosomal vesicles. Endosomal trafficking is controlled by a variety of adaptor proteins [6]. Most often, the receptors recycle back to the cell surface in recycling endosomes, and ligands are routed towards lysosomes for protein degradation. The panel of ligands for endocytic membrane receptors include cell adhesion molecules, signaling molecules, and nutrients. Therefore, the endocytic apparatus is involved in regulating cell adhesion and migration, cell signaling, and cell metabolism [5,6]. Multiple endocytic proteins are dysregulated in cancer [7]. In particular, studies have highlighted the importance of adaptor proteins (e.g., AP2, clathrin, dynamin, and the Rab subfamily of proteins) in regulating cancer cell migration and invasion [7,8,9,10]. However, less is known about the role of endocytic membrane receptors in cancer [7]. 

LRP2 (megalin) is a large endocytic receptor (600 kDa) highly expressed in specialized absorptive epithelia, such as the proximal tubules of the kidney, the thyroid gland, and glandular epithelium of the breast. LRP2 has more than 40 identified ligands, including lipoproteins, albumin, vitamin carrier proteins, hormones and signaling molecules, enzymes and enzyme inhibitors, and immunoglobulins [11,12,13,14,15,16]. LRP2 is most studied in the proximal tubules of the kidney, where it is essential for the recovery of proteins, nutrients, and minerals from glomerular ultrafiltrate [11,12]. Accordingly, patients diagnosed with multifaceted Donnai–Barrow/Facio-Ocular-Acustico-Renal Syndrome, a rare autosomal recessive disorder resulting from mutations in the *LRP2* gene, display low-molecular-weight proteinuria, along with a panel of other disorders [17,18]. 

In contrast to the restricted expression pattern of LRP2 in adults, LRP2 is more widely expressed during embryonic development [19,20]. LRP2 is required for proper development of numerous fetal tissues, including the brain, as is evident from the severe brain malformations observed in LRP2 knockout mice and in patients diagnosed with Donnai–Barrow syndrome [17,18,21,22,23,24]. 

Despite the established importance of LRP2 in many tissues throughout embryonic development and in absorptive epithelia in adults, the role of LRP2 in tumors that arise from these tissues is not well understood, and only a few studies have addressed this. LRP2 has been proposed as a potential target for selective anti-cancer drug delivery, with studies showing that kidney-targeting multimodal micelles [25] and light-chain conjugated nanoparticles [26] can target renal cell carcinoma cells through their interaction with LRP2. LRP2 expression has been observed in breast cancer cell lines, where LRP2 has been suggested to facilitate vitamin D uptake [27,28]. Finally, it has been reported that LRP2 is expressed in a subset of melanoma cell lines and melanoma tumors, and that LRP2 in melanoma cells is involved in receptor-mediated endocytosis [29].

In the present study, we examine the gene expression of *LRP2* across 33 cancer types in The Cancer Genome Atlas. As expected, *LRP2* was highly expressed in tumors that arise from absorptive epithelia, such as clear cell renal cell carcinoma, papillary renal cell carcinoma, mesothelioma, invasive breast carcinoma, and papillary thyroid cancer. However, in a subset of tumors from these cancer types, we observed epigenetic silencing of *LRP2*. *LRP2* expression was inversely correlated to methylation of a specific CpG site in the first intron (cg02361027) of the *LRP2* gene. Interestingly, low *LRP2* expression was associated with tumor cell dedifferentiation and poor outcome in clear cell renal cell carcinoma, papillary renal cell carcinoma, mesothelioma, papillary thyroid carcinoma, and invasive breast carcinoma.

## 2. Materials and Methods

### 2.1. Tissue

Sections of formalin-fixed, paraffin-embedded (FFPE) breast cancer samples from patients diagnosed with invasive ductal carcinoma from 2010 to 2014 were collected from the archives at Department of Pathology, Aarhus University Hospital, Denmark. Following surgical resection, breast cancer samples were fixed immediately in 4% formalin for 24 to 48 h, dehydrated in graded ethanol, and embedded in paraffin. 

Invasive ductal carcinoma samples were included based on receptor status, proliferation rate, histological grade, and TNM classification. Inclusion criteria for the 12 Luminal A subtype samples included in this study were as follows: estrogen receptor (ER) positivity ≥ 80%; normal human epidermal growth factor receptor (HER2) status; and Ki67 ≤ 10%. The clinical-pathological parameters were as follows: histological grade = I for all; metastases = 0 for all; average tumor size = 14.4 mm; and age = 53–81 years (average 64 years). All samples were obtained from patients that had not been assigned neoadjuvant therapy. 

Healthy human breast gland epithelium samples were obtained from breast reduction surgeries performed from 2012 to 2014 at Aarhus University Hospital, Denmark and were treated the same way as the breast cancer samples. Samples from patients between 24–45 years of age (average 35 years) at the time of reductive plastic surgery and without previously known cancer were included.

Human kidney cortex samples were collected from the archives at the Department of Pathology, Aarhus University Hospital. These tissue samples were isolated during postmortem autopsies and originated from individuals with no sign of kidney disease. These tissue samples were treated the same way as the breast tissue samples in terms of fixation and paraffin embedding.

Before the use of any biological material, each patient’s wish regarding the use of his or her tissue sample for research was inspected using the Tissue Application Registry in Denmark. All identifiers were removed after selection of tissue samples from the archives according to the guidelines from the Regional Ethical Committee (Region of Central Denmark). This study was approved by the Regional Ethical Committee (case#: 1-10-72-341-15) and by the Danish Data Protection Agency (case#: 1-16-02-678-15).

### 2.2. Antibodies

A previously characterized polyclonal rabbit anti-human LRP2 antibody [30,31,32] was protein G purified and used for immunohistochemistry (working concentration 16.5 μg/mL). In addition, we generated a novel monoclonal mouse anti-human LRP2 antibody against ligand-affinity purified full-length human LRP2 of renal origin using classical hybridoma technology. Hybridoma culture supernatant containing the monoclonal mouse anti-human LRP2 antibody was used for immunohistochemistry (diluted 1:100).

### 2.3. Immunohistochemistry

Sections of 2.5 µm from the FFPE samples of healthy human kidney cortex, healthy breast gland epithelium, and invasive ductal carcinoma samples were cut using a Leica RM2165 rotary microtome (Leica, Ballerup, Denmark), mounted on positive charged Superfrost^®^ glass slides (Thermo Scientific, Slangerup, Denmark), and dried for 1 h at 60 °C.

Sections of healthy human kidney cortex were mounted alongside sections of breast tissue on all slides. Kidney cortex was included as a positive control for LRP2 labeling, as the expression of LRP2 in human kidney cortex is well characterized [33].

Immunohistochemical staining was performed using a Ventana XT Benchmark automated staining system (Ventana Medical Systems, Roche, Tucson, AZ, USA). Heat-induced epitope retrieval (HIER) was performed at pH 9. Visualization of LRP2 labeled with monoclonal mouse anti-human LRP2 was performed using the OptiView diaminobenzidine (DAB) detection kit (#760-700, Ventana Medical Systems, Roche), and visualization of LRP2 labeled with polyclonal rabbit anti-human LRP2 was performed using the UltraView DAB detection kit (#760-500, Ventana Medical Systems, Roche), including an additional UltraWash step. Sections were incubated with primary antibodies for 30 min. All sections were counterstained with hematoxylin. Sections were dehydrated and mounted with xylene and film using a Tissue-Tek SCA film coverslipper (Sakura Finetek, Zoeterwoude, The Netherlands). Whole slide images were captured using a Nanozoomer 1.0-HT scanner (Hamamatsu Photonics K.K., Hamamatsu City, Japan) with a magnification of 20×. Slides were analyzed using NDP viewer (Hamamatsu Photonics K.K.).

### 2.4. Data Availability

The Cancer Genome Atlas (TCGA) Pan-Cancer datasets were downloaded from the UCSC Xena data portal (https://xena.ucsc.edu/, accessed on 4 November 2021). TCGA Pan-Cancer RNA sequencing data were processed using the Toil pipeline [34], which uses STAR [35] to generate alignments (reference genome GRCh38) and performs quantification using RSEM [36] and Kallisto [37]. RNA sequencing data were downloaded as RSEM-based normalized gene quantifications and then log2-transformed with an offset of 1. TCGA Pan-Cancer Illumina450K methylation data were available as CpG β values. TCGA Pan-Cancer survival data were reviewed and made available by the TCGA Pan-Cancer Clinical Data Resource [38]. Single-cell data from renal cell carcinoma [39] and breast cancer [40] were accessed and visualized using the Broad Single Cell Portal (https://singlecell.broadinstitute.org/single_cell, accessed on 1 October 2022). Proteomics data from Clinical Proteomic Tumor Analysis Consortium (CPTAC) were retrieved as processed gene level values using the Python package cptac [41]. Processed data from the Molecular Taxonomy of Breast Cancer International Consortium (METABRIC) [42] were available from cBioportal (https://www.cbioportal.org/study/summary?id=brca_metabric, accessed on 19 November 2021). Mesothelioma gene-expression data from Bott et al. is available at the Gene Expression Omnibus (GEO) under accession number GSE29354 and clinical data are available from the original publication [43]. Mesothelioma gene-expression data from Gordon et al. is available from the GEO under accession number GSE2549 and clinical data are available from the original publication [44].

### 2.5. Single-Cell RNA Sequencing Analysis

Single-cell data from renal cell carcinoma [39] and breast cancer [40] were accessed using the Broad Single Cell Portal. UMAPs color-coded for cell annotations or normalized LRP2 expression values were generated in the portal (subsampling 10.000 cells). Violin plots and dot plots of LRP2 expression across major and minor cell types in breast cancer were generated using Seurat [45].

### 2.6. LRP2 Survival Analysis

For TCGA survival analyses, we used the recommended survival parameters from the TCGA Pan-Cancer Clinical Data Resource [38]. Survival analysis was not performed for seven cancer types flagged as having insufficient data for robust analysis. Univariate and multivariate Cox proportional-hazards modelling was performed using the coxph function from the survival R package (https://cran.r-project.org/web/packages/survival/index.html, accessed on 8 January 2023). Statistical analysis for the model was performed using likelihood-ratio test. Survival curves for *LRP2*^high^ and *LRP2*^low^ groups were plotted using the Kaplan–Meier method. For each cancer type, the cohort was stratified based on the lower quartile of *LRP2* expression. Statistical analysis of survival curve differences between groups was performed using the log-rank test. We also performed survival analysis using the KMplotter online tool (https://kmplot.com/analysis/, accessed on 29 October 2022), which is an integrated database for transcriptomic datasets [46]. *LRP2*-based survival analysis was performed on the breast cancer microarray dataset, using a lower quartile cutoff for stratification and the overall survival parameter.

### 2.7. LRP2 Methylation Analysis

TCGA Pan-Cancer Illumina450K methylation β values were downloaded from the UCSC Xena data portal. Illumina450K probes mapping to the *LRP2* gene were extracted from the Illumina450K manifest file (HM450.hg38.manifest.gencode.v36), available from the Genomic Data Commons portal (https://gdc.cancer.gov/about-data/gdc-data-processing/gdc-reference-files, accessed on 18 February 2023). There were 19 CpG sites with available data mapped to the *LRP2* gene. The location of CpG sites in relation to the full-length *LRP2* transcript (ENST00000649046) were determined using exon and intron intervals available from ENSEMBL GRCh38.

### 2.8. Differential Gene Expression and Gene Set Enrichment Analysis

Differential gene-expression analysis between groups was performed using limma-voom [47,48] within the Xena Differential Gene Expression Analysis Pipeline (analysis.xenahubs.net, accessed on 20 February 2023). *LRP2*^high^ and *LRP2*^low^ groups were defined using the lower quartile cutoff of *LRP2* expression within each cancer type. Gene set enrichment analysis (GSEA) was performed on the ranked gene list from differential gene-expression analysis (*p* value x direction of fold change) using the fgsea (biorxiv.org/content/10.1101/060012v3, accessed on 8 January 2023) package in R. Gene sets included HALLMARKS, GO Biological Process (BP), GO Cellular Component (CC), and GO Molecular Function (MF). Enrichment plots were generated using clusterProfiler [49,50].

### 2.9. LRP2 Tumor Differentiation Analysis

For thyroid cancer, the tumor differentiation score was calculated as the average expression of 16 genes related to thyroid function and metabolism (DIO1, DIO2, DUOX1, DUOX2, FOXE1, GLIS3, NKX2-1, PAX8, SLC26A4, SLC5A5, SLC5A8, TG, THRA, THRB, TPO and TSHR) as previously defined [51]. Breast cancer PAM50 subtypes were available from the METABRIC sample annotation file (cBioPortal). Mesothelioma histological subtypes were available from the TCGA MESO sample annotation file (UCSC Xena data portal).

### 2.10. Statistical Analysis and Data Visualization

Data handling, statistical analysis, and data visualization were performed in Prism version 9.2.0 and R version 4.0.4. Heatmaps were generated using the ComplexHeatmap package (https://github.com/jokergoo/ComplexHeatmap, accessed on 8 January 2023) in R. Boxplots and scatterplots were generated using the ggplot2 [52] and ggpubr [53] packages in R.

## 3. Results

### 3.1. LRP2 Expression across Human Cancers

**Figure 1:**
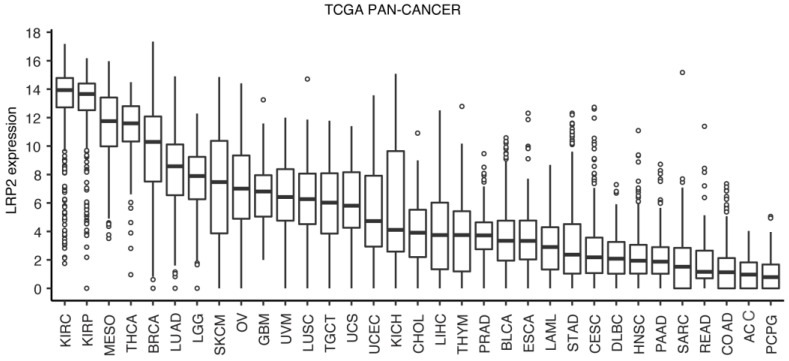
*LRP2* expression across human cancers. Boxplot of *LRP2* expression (RSEM norm_count log2 transformed with offset of 1) across each cancer type in the TCGA Pan-Cancer dataset. Boxplot lines represent lower quartile, median, and upper quartile. Whiskers represent 1.5 times above or below interquartile range. Points reflect outliers. Full list of cancer type abbreviations is provided in Table S1.

To characterize the expression of *LRP2* across human cancers, we accessed RNA sequencing data from TCGA, consisting of 10,534 samples across 33 cancer types. *LRP2* expression was variable across cancer types with the highest expression in cancers arising from tissues known to express *LRP2* in the healthy state (Figure 1). This included renal cell carcinoma (KIRC) and papillary renal cell carcinoma (KIRP) originating from proximal tubule epithelial cells, invasive breast carcinoma (BRCA) with origin in breast epithelial cells, and papillary thyroid carcinoma (THCA) that derives from thyroid epithelial cells. Lung cancer types, including mesothelioma (MESO), lung adenocarcinoma (LUAD), and lung squamous cell carcinoma (LUSC), also showed mid-to-high levels of *LRP2*, which is consistent with the reported expression of LRP2 in pleural mesothelial cells in the embryo [19] and in type II pneumocytes in the adult lung [54]. Moderate levels of *LRP2* were detected in low-grade glioma (LGG) and glioblastoma multiforme (GBM), although there have only been a few reports of LRP2 expression in the brain and spinal cord [55,56,57,58]. Furthermore, these data confirmed mid-to-high *LRP2* expression in a subset of cutaneous melanoma (SKCM), which is consistent with our previous study [29].

### 3.2. Tumor LRP2 Expression Is Restricted to Malignant Cells and Correlates with LRP2 Protein Levels

**Figure 2:**
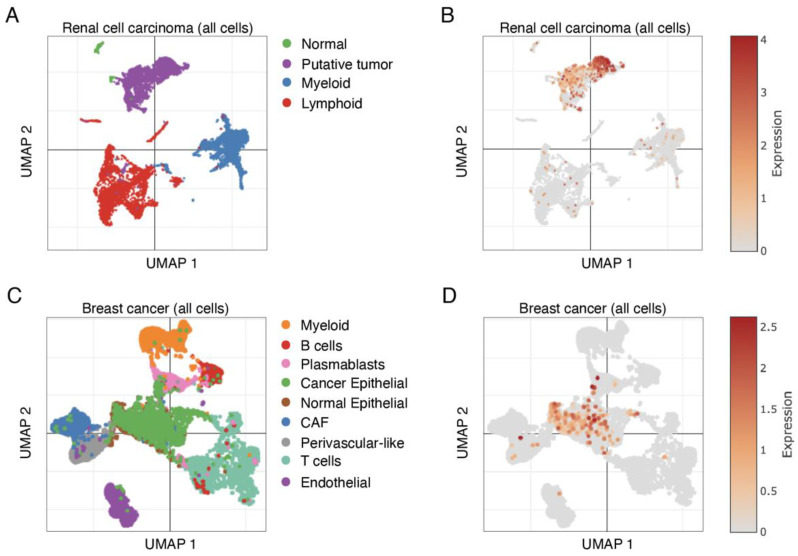
*LRP2* expression is largely restricted to malignant cells in the tumor microenvironment. (**A**) UMAP visualization of single-cell RNA sequencing data integrated across 8 advanced renal cell carcinoma tumors (subsampling 10.000 cells) and colored by lineage. (**B**) Log-normalized expression of *LRP2* overlaid on UMAP from panel A. (**C**) UMAP visualization of single-cell RNA sequencing data integrated across 26 primary breast tumors (subsampling 10.000 cells) and colored by major cell type. (**D**) Log-normalized expression of *LRP2* overlaid on UMAP from panel C. CAF: cancer associated fibroblasts.

*LRP2* expression in bulk RNA sequencing samples could potentially be derived from multiple cell types in the tumor microenvironment. Single-cell RNA sequencing data from clear cell renal cell carcinoma [39] and breast cancer [40], two cancer types with high expression of *LRP2*, revealed that *LRP2* expression is restricted to epithelial cancer cells (and residual normal epithelial cells) in the tumor microenvironment (Figure 2 and Figure S1A–D). Lack of *LRP2* expression in stromal and immune cells is consistent with our previous immunohistochemical evaluation of melanoma tumors [29].

Gene expression and corresponding protein levels correlate to varying degrees in cancer cells and tumors [59]. We compared gene-level paired transcriptomic and proteomic data for clear cell renal cell carcinoma and invasive breast carcinoma in the Clinical Proteomic Tumor Analysis Consortium (CPTAC) [60,61]. In both KIRC (Pearson’s r = 0.83, *p* < 0.0001, Figure S2A) and BRCA (Pearson’s r = 0.58, *p* < 0.0001, Figure S2B), we observed a strong correlation between LRP2 gene and protein levels. Thus, *LRP2* levels measured in tumor bulk RNA sequencing data reflect tumor-specific expression and correlate with protein abundance.

### 3.3. Confirmation of LRP2 Protein Expression in Luminal Invasive Breast Carcinoma

To confirm protein expression of LRP2 in a cancer of epithelial origin, we performed immunohistochemical analysis of sections of 12 formalin-fixed paraffin-embedded luminal A invasive breast carcinomas. 

We used a previously characterized polyclonal rabbit anti-human LRP2 antibody [30,31,32] and a novel monoclonal mouse anti-human LRP2 antibody. We confirmed that the monoclonal anti-human LRP2 antibody reacted with LRP2 by Western blotting and by immunohistochemical staining of sections of kidney cortex, which revealed a staining pattern similar to that of the polyclonal anti-human LRP2 antibody (Figure S3).

First, immunohistochemical examination of sections from four different healthy glandular breast tissue samples showed that LRP2 was expressed exclusively in breast epithelial cells, where it localizes to the luminal surface (Figures S4 and S5), similar to its expression pattern in kidney proximal tubule cells.

**Figure 3:**
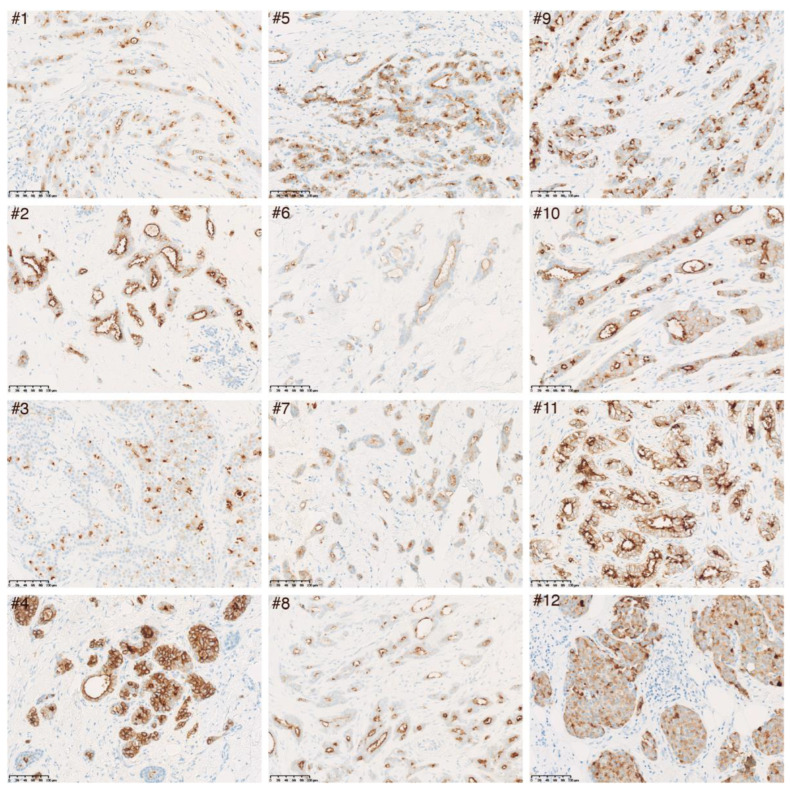
Immunohistochemical analysis of LRP2 protein expression in 12 luminal A invasive ductal carcinomas. Representative images of sections from 12 different invasive ductal carcinomas labeled with a monoclonal mouse anti-human LRP2 antibody (1:100) are shown. Magnification: 20×. Scale bar: 100 µm. Similar images from labeling of sections from the same 12 luminal A invasive ductal carcinomas with a polyclonal rabbit anti-human LRP2 antibody are shown in Figure S6.

Next, immunohistochemical analysis of 12 luminal A invasive breast cancer samples was performed using either the monoclonal mouse anti-human LRP2 antibody (Figure 3) or the polyclonal rabbit anti-human LRP2 antibody (Figure S6). LRP2 was detectable, although to a varying degree, across all 12 tumor samples. LRP2 protein expression appeared confined to malignant cells (and potentially residual normal epithelial cells), which is consistent with the single-cell RNA sequencing data presented in Figure 2C,D. LRP2 was observed both at the plasma membrane and in cytoplasmic compartments distributed throughout malignant cells.

### 3.4. Low LRP2 Expression Is Associated with Poor Survival in Multiple Cancers

**Figure 4:**
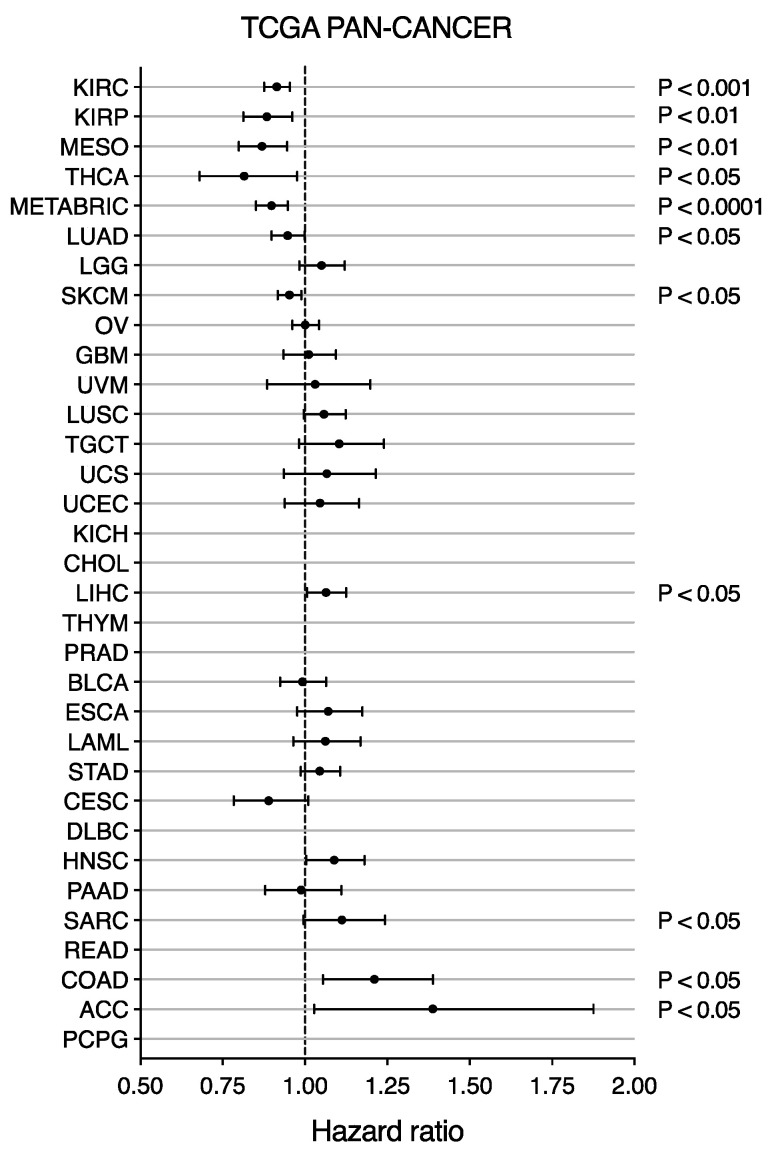
Low *LRP2* expression is associated with poor survival in multiple cancers. *LRP2* expression hazard ratio with 95% confidence intervals is shown for each cancer type. Hazard ratio < 1 indicates decreased risk with higher *LRP2*. Hazard ratio > 1 indicates increased risk with higher *LRP2*. Cancer types are ranked (top to bottom) according to their median expression of *LRP2*. *p* value from the likelihood-ratio test is shown for significant cancer types. No data are shown for seven cancer types where analysis was not performed due to insufficient event number. Full list of cancer-type abbreviations is provided in Table S1. Source data provided in Table S2.

Investigation of *LRP2* levels across cancer types showed considerable variation in *LRP2* expression within individual cancer types (Figure 1), which could suggest that *LRP2* is deregulated in a subset of tumors. This observation prompted us to test the association between *LRP2* expression and clinical outcome. To do so, we performed univariate Cox proportional-hazards modelling of *LRP2* expression and survival within individual TCGA cancer types (Figure 4). Survival parameters were chosen based on the recommendations from the TCGA Pan-Cancer Clinical Data Resource and analysis was not performed for seven tumor types flagged as having an insufficient event number [38]. Breast cancer survival analysis was performed using the METABRIC cohort, which includes long-term clinical follow-up data for > 2,000 patients [42]. Interestingly, low levels of *LRP2* were associated with poor patient outcome in seven of the eight cancer types that express the highest levels of *LRP2* on average (Figure 4, Table S2), including kidney renal clear cell carcinoma (KIRC) (HR = 0.91, *p* < 0.001), kidney renal papillary cell carcinoma (KIRP) (HR = 0.88, *p* < 0.01), mesothelioma (MESO) (HR = 0.87, *p* < 0.01), thyroid carcinoma (THCA) (HR = 0.82, *p* < 0.05), breast cancer (METABRIC) (HR = 0.90, *p* < 0.0001), lung adenocarcinoma (LUAD) (HR = 0.95, *p* < 0.05), and skin cutaneous melanoma (SKCM) (HR = 0.95, *p* < 0.05).

**Figure 5:**
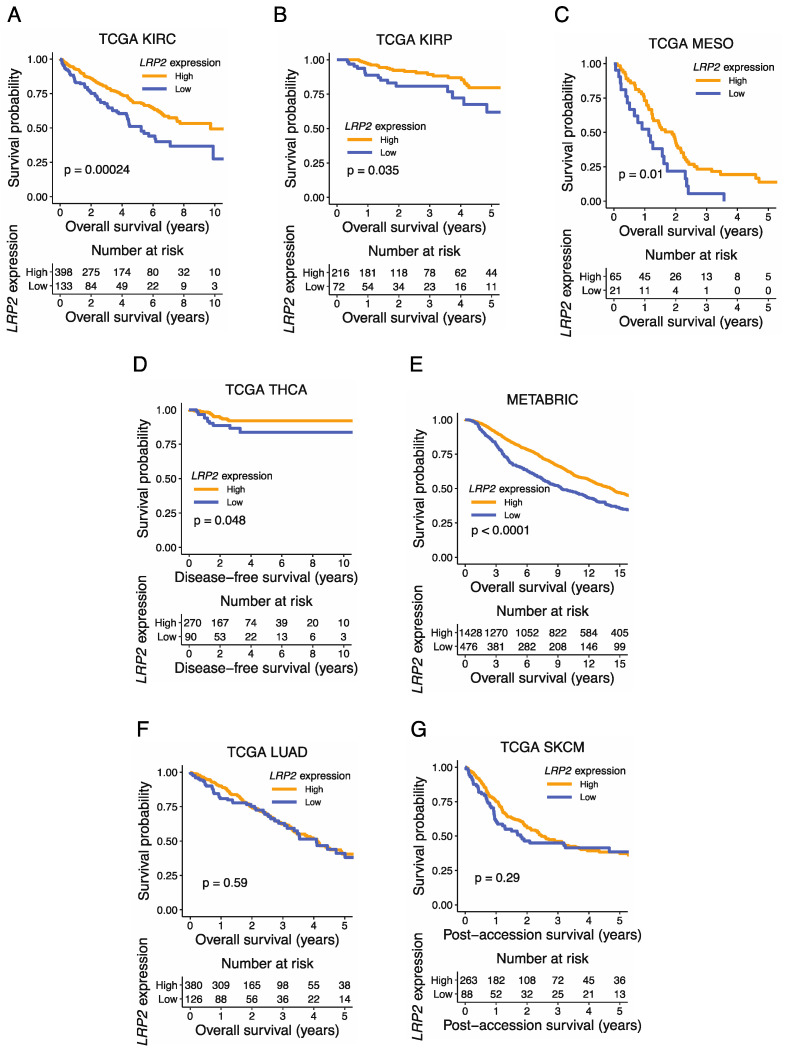
Low *LRP2* expression is associated with poor outcome in multiple cancers. (**A**–**G**) Kaplan–Meier curve of *LRP2*^high^ and *LRP2*^low^ groups stratified based on the lower quartile of *LRP2* expression in each dataset (upper panel) and risk table (lower panel). *p* value from the log-rank test is shown.

Based on the observation that a subset of tumors within these cancer types showed lower *LRP2* expression, we stratified samples into *LRP2*^high^ or *LRP2*^low^ based on a lower quartile cutoff and performed Kaplan–Meier analysis. As expected, this provided similar results as the Cox proportional-hazards model and showed significantly poorer survival of patients with *LRP2*^low^ tumors in KIRC (*p* = 0.00024), KIRP (*p* = 0.035), MESO (*p* = 0.01), THCA (*p* = 0.048), and METABRIC (*p* < 0.0001) (Figure 5A–E). However, at this particular cutoff, there were no significant differences between the survival of patients with *LRP2*^high^ or *LRP2*^low^ tumors in LUAD (*p* = 0.59) and SKCM (*p* = 0.29)(Figure 5F,G).

We sought to validate the association between low *LRP2* and poor outcome in additional cohorts. In a previous unbiased analysis of primary clear cell renal cell carcinomas, low *LRP2* was identified as one of 259 genes that predicted poor outcome after surgery [62]. Low *LRP2* was also associated with shorter overall survival in the KMplotter database (lower quartile cutoff Kaplan–Meier analysis, HR = 0.64, *p* < 0.0001, Figure S7A), which is an integrated database of breast cancer gene-expression data [46]. Likewise, low *LRP2* was associated with poor outcome in the Bott et al. microarray cohort for mesothelioma (univariate Cox proportional-hazards model, HR = 0.81, *p* = 0.038) and trended towards significance in the Gordon et al. microarray cohort for mesothelioma (univariate Cox proportional-hazards model, HR = 0.88, *p* = 0.108) (Figure S7C) [43,44]. 

Based on these analyses, we considered a framework where *LRP2* expression is generally maintained in the cancer setting, but where its expression is lost in a subset of tumors, and that this is associated with poor patient outcome.

### 3.5. Low LRP2 Expression in Human Cancer Is Associated with First Intron CpG Methylation

To characterize the mechanisms that lead to putative loss of *LRP2* expression in some tumors, we next explored the mutation profile of *LRP2* in human cancers. Loss-of-function mutations and gene deletions in *LRP2* are rare and cannot not explain the extent of lost *LRP2* expression observed across cancer types. Therefore, we hypothesized that epigenetic events could account for loss of *LRP2* expression in cancer.

**Figure 6:**
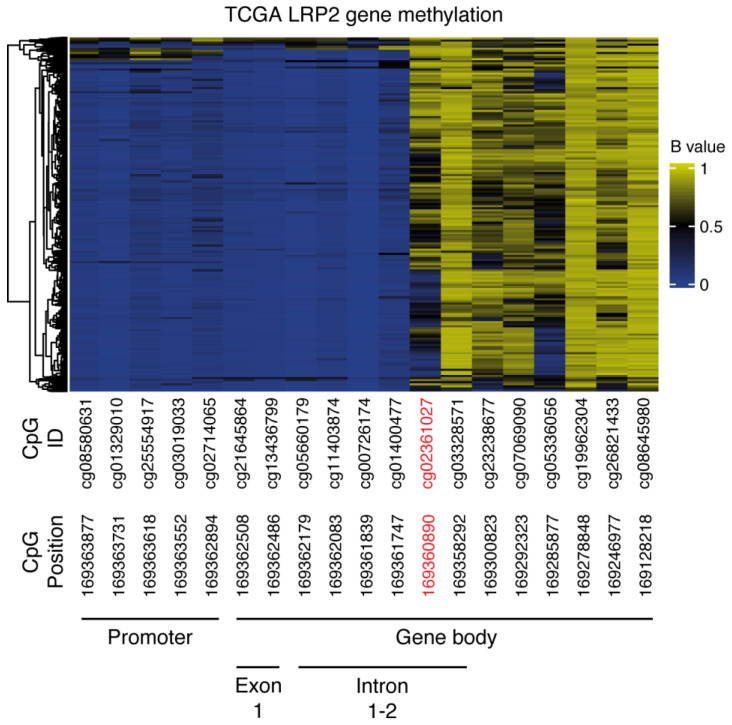
CpG methylation in the *LRP2* gene locus. Heatmap of CpG site values from the 19 CpG sites in the *LRP2* gene included in the Illumina450K TCGA Pan-Cancer dataset. *LRP2* β values for each CpG site in KIRC, KIRP, MESO, THCA, BRCA, LUAD, and SKCM are shown. CpG ID, CpG position on chromosome 2 (GRCh38) and location relative to the full-length *LRP2* transcript (ENST00000649046) are shown below the heatmap. cg02361027 at position 169360890, which shows a strong inverse correlation to *LRP2* expression, is highlighted in red.

To investigate this, we accessed TCGA Pan-Cancer methylation data from the Illumina 450K array, which provides a fraction of methylation values (β values) for 450,000 CpG sites in the human genome [63]. There were 19 probes available in the *LRP2* promoter and gene body in the dataset. Heatmap visualization showed that CpG sites in the promoter region of *LRP2* were demethylated, while CpG sites in the gene body were largely methylated (Figure 6, Table 1). Consistent with the general understanding of how DNA methylation regulates gene expression, CpG methylation in the *LRP2* promoter region was negatively correlated to *LRP2* expression, while sites in the gene body mainly showed a positive correlation (Table 1).

Of interest, a single CpG site in the first intron of *LRP2*, cg023161027, showed differential methylation across tumors and a strong inverse correlation with *LRP2* expression (Pearson’s R = −0.65, *p* < 1 × 10^−^^100^, Table 1). cg02361027 is found at position 169360890 (human genome version hg38) on the + strand of chromosome 2 and is in close proximity to a CpG island, stretching from position 169361804-169363035. Its negative correlation to *LRP2* expression is consistent with studies showing genome-wide inverse correlations between first intron methylation and gene expression [64,65].

**Figure 7:**
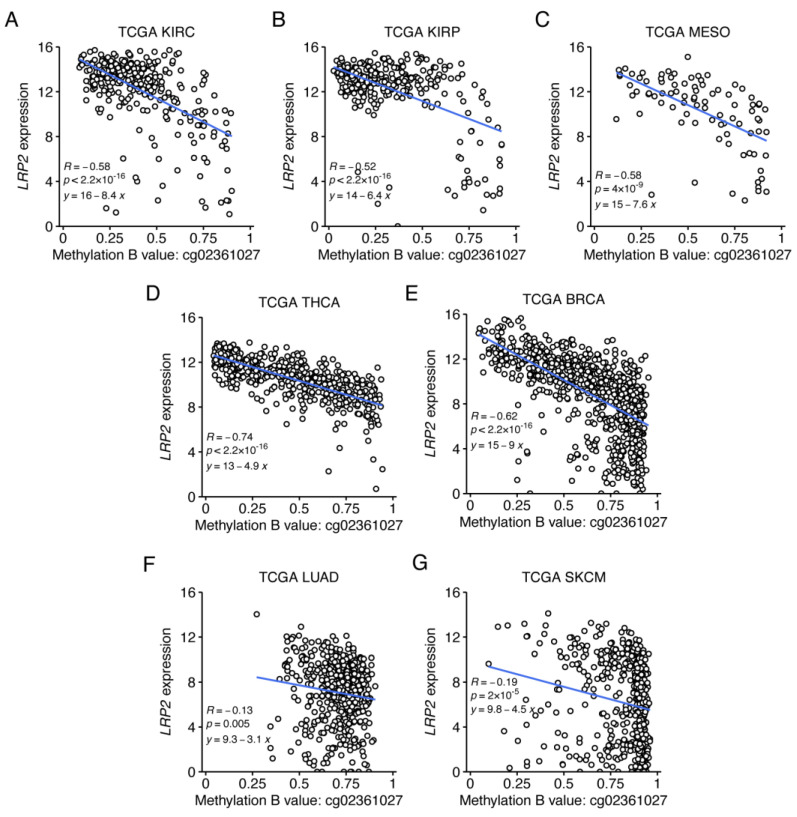
*LRP2* silencing is associated with methylation of a specific CpG site in the first intron. (**A**–**G**) Scatter plots of *LRP2* expression (RSEM norm_count log2 transformed with offset of 1) versus methylation β values for the CpG site cg02361027. Pearson’s R and corresponding *p* values are shown together with the linear regression line and equation.

To further explore the relationship between *LRP2* expression and cg02361027 methylation, we calculated their correlation within individual cancer types (Figure S8). This showed that the negative correlation is strongest in tumor types that on average express the highest levels of *LRP2* (KIRC, KIRP, MESO, THCA and BRCA) (Figure 7A–E). On the other hand, the correlation is modest within tumor types that express mid-to-low levels of *LRP2*, including LUAD and SKCM (Figure 7F,G), suggesting that other factors contribute to differential *LRP2* expression in these cancer types.

### 3.6. LRP2 Silencing Is Associated with Tumor Dedifferentiation

**Figure 8:**
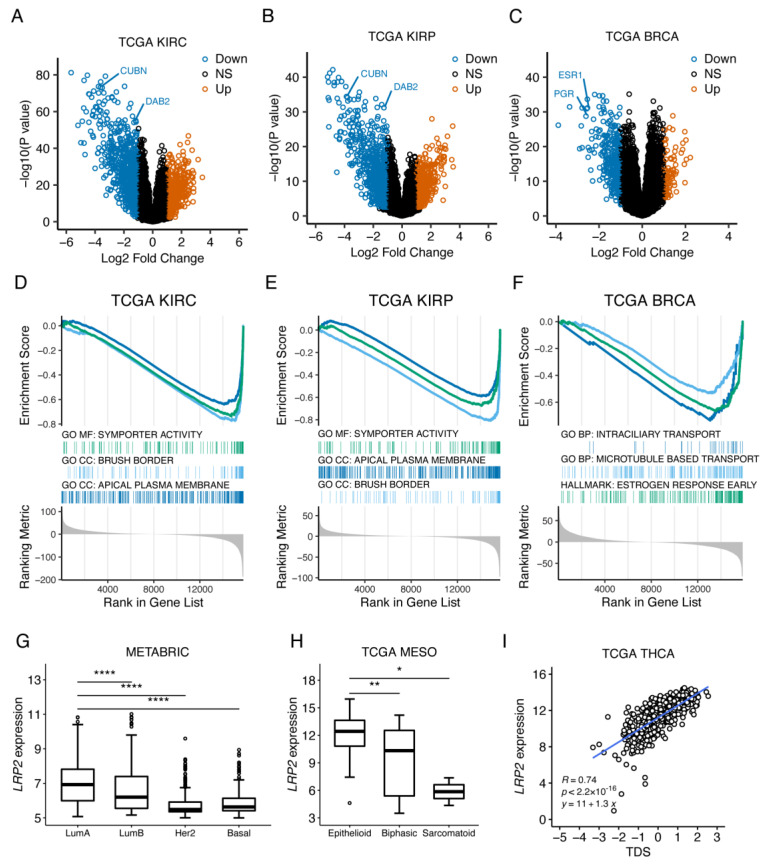
*LRP2* silencing is associated with tumor dedifferentiation. (**A**–**C**) Volcano plot of differential gene-expression analysis of *LRP2*^low^ versus *LRP2*^high^ tumors (lower quartile cutoff) in KIRC (panel A), KIRP (panel B), and BRCA (panel C). Genes downregulated in *LRP2*^low^ tumors (FDR < 0.01 and log2 fold change < −1) are shown in blue. Genes upregulated in *LRP2*^low^ tumors (FDR < 0.01 and log2 fold change > 1) are shown in orange. Non-significant genes (FDR > 0.01) are shown in black. (**D**–**F**) Enrichment plots from gene set enrichment analysis showing selected top pathways downregulated in *LRP2*^low^ tumors in KIRC (panel D), KIRP (panel E), and BRCA (panel F). (**G**) Boxplot of *LRP2* expression across PAM50 molecular subtypes in breast cancer cohort METABRIC. (**H**) Boxplot of *LRP2* expression (RSEM norm_count log2 transformed with offset of 1) across histological subtypes in TCGA MESO. Boxplot lines represent lower quartile, median, and upper quartile. Whiskers represent 1.5 times above or below interquartile range. Points reflect outliers. (**I**) Scatter plot of correlation between *LRP2* expression (RSEM norm_count log2 transformed with offset of 1) and 16-gene tumor differentiation score in TCGA THCA. Pearson’s R and corresponding *p* values are shown together with the linear regression line and equation. Wilcox rank-sum test: * *p* ≤ 0.05, ** *p* ≤ 0.01, **** *p* ≤ 0.0001. TDS: tumor differentiation score.

LRP2 expression in adults is restricted to absorptive epithelia, and LRP2 is considered a marker for proximal tubule epithelial cell-differentiation status. Based on this, we hypothesized that the loss of *LRP2* expression observed in some tumors is associated with dedifferentiation. Differential gene-expression analysis between *LRP2*^high^ and *LRP2*^low^ tumors in KIRC and KIRP revealed that multiple genes (including *CUBN* and *DAB2* encoding functional LRP2 partners in proximal tubule epithelial cells) (Figure 8A,B, Table S3) and pathways related to specialized proximal tubule epithelial cell functions (Figure 8D,E, Table S4) were also downregulated in *LRP2*^low^ tumors. Similar analysis for BRCA revealed downregulation of multiple genes (including genes encoding hormone receptors ESR1 and PGR) (Figure 8C, Table S3) and pathways related to mammary gland epithelium differentiation and function (e.g., estrogen response) (Figure 8F, Table S4) in *LRP2*^low^ tumors.

We further evaluated the relationship between *LRP2* expression and molecular subtypes or signatures related to differentiation in breast cancer, mesothelioma, and thyroid cancer. In breast cancer, tumors are classified using the PAM50 gene signature into four molecular subtypes (luminal A, luminal B, HER2-enriched, and basal), which differ in their biological characteristics and prognosis [66]. Luminal tumors show a higher level of epithelial differentiation and estrogen receptor expression and signaling, while HER2 and basal tumors are more dedifferentiated [67]. Consistent with higher expression of *LRP2* in more epithelial-like tumors, *LRP2* expression was significantly higher in luminal A tumors compared to luminal B (*p* < 0.0001), HER2 (*p* < 0.0001), and basal (*p* < 0.0001) tumors in the METABRIC cohort (Figure 8G). In mesothelioma, histological subtypes are also classified as epithelioid, biphasic, or sarcomatoid based on their differentiation state [68]. In the TCGA MESO dataset, *LRP2* expression was higher in epithelioid tumors compared to biphasic (*p* < 0.01) and sarcomatoid (*p* < 0.05) tumors (Figure 8H). In papillary thyroid cancer, a 16-gene tumor differentiation score (TDS) correlated to patient prognosis was defined by the TCGA THCA working group [51]. *LRP2* showed a strong correlation to TDS in the TCGA THCA dataset (Pearson’s R = 0.76, *p* < 2.2 × 10^−^^16^) (Figure 8I). These examples provide evidence that *LRP2* silencing is related to a dedifferentiated tumor state in various epithelial cancer types.

Overall, this explorative analysis of the TCGA dataset is consistent with a model where epigenetic silencing of *LRP2* is associated with tumor dedifferentiation and poor patient outcome in multiple cancer types arising from LRP2-expressing absorptive epithelia.

## 4. Discussion

To investigate the clinical and molecular correlates of *LRP2* expression across human cancers, we performed an integrated analysis of publicly available data from The Cancer Genome Atlas. *LRP2* was highly expressed in cancers that arise from absorptive epithelia, such as clear cell renal cell carcinoma, papillary renal cell carcinoma, mesothelioma, invasive breast carcinoma, and papillary thyroid cancer. A subset of tumors within these cancer types show downregulation of *LRP2*, which is correlated with epigenetic alterations in the *LRP2* gene locus, tumor dedifferentiation, and poor patient outcome.

To our knowledge, there have been no earlier systematic studies of *LRP2* in cancer. We have previously demonstrated that LRP2 gene and protein expression is acquired in a subset of melanoma tumors [29]. However, our pan-cancer analysis did not immediately reveal other cancer types with gained expression of *LRP2*. Instead, our data support a model where *LRP2* is maintained in cancers arising from absorptive epithelial cells known to express high levels of *LRP2* in the healthy state. Our data also highlight deregulation of *LRP2* as a common event in these cancer types.

One limitation of our study is that it leveraged bulk RNA sequencing data, which could be confounded by expression of *LRP2* in non-malignant cell types and might not reflect functional protein levels. To address this limitation, we queried single-cell RNA sequencing data and found that *LRP2* expression is restricted to malignant cells. Furthermore, we used recent proteomics data from human tumors to demonstrate a strong correlation between *LRP2* transcript and protein levels. Therefore, *LRP2* levels in tumor bulk RNA measurements can be considered to reflect tumor-specific expression and to correlate with protein abundance.

Additionally, we confirmed LRP2 protein expression in healthy breast epithelial cells and malignant cells of 12 different invasive breast carcinomas. LRP2 expression was confined to the apical plasma membrane in healthy breast epithelial cells but was observed both at the plasma membrane and in cytoplasmic compartments distributed throughout malignant cells. We speculate that this could reflect increased endocytic activity and thus intracellular trafficking of LRP2 in malignant cells as opposed to healthy cells.

*LRP2* in epithelial cancers was strongly and inversely correlated to methylation of a specific CpG site (cg02361027) in the first intron of the *LRP2* locus. To our knowledge, the present study is the first identification of a robust correlation between *LRP2* levels and DNA methylation in cancer. The inverse correlation between *LRP2* expression and cg02361027 methylation could suggest that this region of the first intron is involved in transcriptional regulation, although this remains to be experimentally proven. Previous studies have demonstrated a genome-wide inverse correlation between first intron methylation and gene expression [64,65] and shown that conserved regions of the first intron are likely to regulate transcription [69]. Indeed, cg02361027 resides in a highly conserved region of the *LRP2* first intron (UCSC Genome Browser, Figure S9). Further studies should determine the correlation between *LRP2* expression and first intron methylation in other biological systems where *LRP2* levels are variable, such as throughout embryonic development [19,20] or in the settings of acute and chronic kidney diseases [70,71,72]. Identification of transcription factors that interact with this region of the *LRP2* locus will also be of interest.

We further observed that *LRP2* downregulation was associated with dedifferentiated tumor subsets in multiple cancer types. We speculate that *LRP2* loss in epithelial cancers might be part of a genome-wide epigenetic reprogramming in epithelial cancers, resulting in coordinated deactivation of epithelial gene expression. However, the results presented in the current study are correlative, and future studies are needed to dissect the exact mechanisms of *LRP2* regulation in cancer.

In the context of cancer, loss of LRP2 protein could alter the cellular uptake and clearance of ligands from the tumor microenvironment. Putative tumor-suppressive functions of LRP2 could be exerted through the clearance of ligands that modulate proliferation, migration, and survival of cancer cells. More complex mechanisms are also possible, where LRP2-mediated clearance of certain molecules from the tumor microenvironment could modulate functions of immune or stromal cells.

Two studies support the role of LRP2 in mediating endocytic uptake of vitamin D (in complex with vitamin D binding protein) and in the activation of vitamin D receptor signaling in breast cancer cells [27,28]. In connection to this, it is important to mention that high serum levels of vitamin D [73,74] and high expression of the vitamin D receptor [75] are linked to better prognosis in breast cancer. In line with this, we find it tempting to speculate that LRP2 also plays a role in this context and that the presence or absence of LRP2 in breast cancer cells would have an important impact on vitamin D homeostasis and receptor signaling. However, this remains to be experimentally proven.

Given that more than 40 ligands have been identified for LRP2 to date [12,16], we hypothesize that multiple tissue-, context- and ligand-dependent cellular signaling pathways would be impacted by downregulation of this receptor in cancer cells.

We show that LRP2 downregulation correlates with poor patient outcome in multiple cancer types, including renal cell carcinoma, mesothelioma, thyroid cancer and invasive breast carcinoma. Interestingly, our analyses also demonstrate that the prognostic value of LRP2 appears to be independent of clinicopathologic variables, such as age, gender, tumor stage, and histological grade (Tables S5–S7). Accordingly, we suggest that future cancer-type-specific studies should investigate further the potential of LRP2 as an independent prognostic biomarker.

In line with the idea that LRP2 might be a potential novel biomarker in certain cancer types, a recent effort to identify genes related to metastasis in premenopausal patients with hormone receptor positive early breast cancer identified low *LRP2* expression as the top independent factor in the Breast Cancer 360 ^TM^ panel [76]. This is consistent with our finding that low *LRP2* predicts poor prognosis in the METABRIC cohort and supports that further studies should be conducted in order to evaluate LRP2 as biomarker in invasive breast carcinomas. It would be natural for such studies to focus on immunohistochemical detection of LRP2, which we previously demonstrated to be useful for the analysis of melanoma tumors [29] and, in the present study, for the analysis of invasive breast carcinomas.

Finally, the findings presented here might also have implications for the use of LRP2 as a target for anti-cancer drug delivery. LRP2 has several features that make it an attractive target for drug delivery. First, LRP2 is a long-lived fast-cycling and recycling endocytic receptor, presumably capable of delivering multiple cytotoxic payloads without being subject to cellular degradation itself [13]. Second, the tissue distribution of LRP2 in adults is limited, and to our knowledge, there are no instances where LRP2 is in direct contact with the bloodstream, limiting the risk of side effects from treatment. Previous studies established LRP2 as an effective target for drug delivery in clear cell renal cell carcinoma [25,26]. Because we show here that high levels of LRP2 are not only observed in clear cell renal cell carcinoma, but also in papillary renal cell carcinoma, mesothelioma, and in invasive breast carcinomas, we speculate that LRP2-targeted drug delivery might be effective in some of these cancer types as well.

## 5. Conclusions

We find that LRP2 expression is largely restricted to cancer types that arise from LRP2-expressing absorptive epithelia. However, we also highlight that a subset of tumors within these cancer types show epigenetic silencing of *LRP2*, which is associated with tumor dedifferentiation and poor survival. These observations now motivate further studies on the biological role of LRP2 in cancer, in particular epithelial cancers, and the potential use of LRP2 as a novel cancer-type-specific prognostic biomarker and target for anti-cancer therapy.

## Figures and Tables

**Table 1:** CpG methylation in the *LRP2* gene locus. CpG IDs and related information from the Illumina450K manifest file. CpG IDs 1 to 2 (from top) are related to a CpG island located on chromosome 2 position 169364262-169364604. CpG IDs 6 to 13 (from top) are related to a CpG island located on chromosome 2 position 169361804-169363035. Pearson’s R and corresponding *p* values between *LRP2* expression and β values for each CpG site in KIRC, KIRP, MESO, THCA, BRCA, LUAD, and SKCM are shown. cg02361027 at position 169360890, which shows a strong inverse correlation to *LRP2* expression, is highlighted in red.

CpG ID	Chr	CpG Position (Start)	Strand	Location Relative to CpG Island	Location Relative to Gene	Pearson r	*p* Value
cg08580631	2	169363877	-	N_Shore	TSS1500	−0.29	4.92 × 10^−61^
cg01329010	2	169363731	+	N_Shore	TSS1500	−0.28	2.49 × 10^−57^
cg25554917	2	169363618	+	S_Shore	TSS1500	−0.33	1.91 × 10^−79^
cg03019033	2	169363552	-	S_Shore	TSS1500	−0.30	1 × 10^−63^
cg02714065	2	169362894	+	Island	TSS1500	−0.30	4.53 × 10^−65^
cg21645864	2	169362508	+	Island	Exon 1	−0.36	2.48 × 10^−95^
cg13436799	2	169362486	+	Island	Exon 1	−0.34	8.43 × 10^−83^
cg05660179	2	169362179	+	Island	Intron 1-2	−0.38	<×10^−100^
cg11403874	2	169362083	+	Island	Intron 1-2	−0.41	<1×10^−100^
cg00726174	2	169361839	-	Island	Intron 1-2	−0.33	5.3 × 10^−78^
cg01400477	2	169361747	+	N_Shore	Intron 1-2	−0.42	<1 × 10^−100^
cg02361027	2	169360890	+	N_Shore	Intron 1-2	−0.65	<1 × 10^−100^
cg03328571	2	169358292	-	N_Shelf	Intron 1-2	0.13	4.95 × 10^−14^
cg23238677	2	169300823	+	OpenSea	Intron 4-5	0.38	<1 × 10^−100^
cg07069090	2	169292323	-	OpenSea	Exon 7	0.31	2.10 × 10^−79^
cg05336056	2	169285877	+	OpenSea	Intron 9-10	−0.03	3.25 × 10^−2^
cg19962304	2	169278848	+	OpenSea	Intron 12-13	0.22	9.24 × 10^−34^
cg26821433	2	169246977	+	OpenSea	Exon 21	0.35	1.63 × 10^−90^
cg08645980	2	169128218	+	OpenSea	Exon 79	0.32	4.27 × 10^−75^

## Data Availability

TCGA Pan-Cancer datasets including RNA sequencing, Illumina 450K methylation and survival data are available at the UCSC Xena data portal (https://xena.ucsc.edu/, accessed on 4 November 2021). Single-cell data from renal cell carcinoma and breast cancer can be accessed and visualized using the Broad Single Cell Portal (https://singlecell.broadinstitute.org/single_cell, accessed on 1 October 2022). Processed gene-level proteomics data from CPTAC can be accessed using the Python package cptac (https://paynelab.github.io/cptac/, accessed on 3 October 2022). Processed data from METABRIC are available from cBioportal (https://www.cbioportal.org/study/summary?id=brca_metabric, accessed on 19 November 2021). Mesothelioma gene-expression data from Bott et al. (GSE29354) and Gordon et al. (GSE2549) are available from GEO. All datasets used in this study are publicly available.

## Supplementary Materials

The following supporting information can be downloaded at: https://www.mdpi.com/article/10.3390/cancers15061830/s1, Figure S1: *LRP2* expression is restricted to epithelial cells in the breast tumor microenvironment. Figure S2: Scatter plot of *LRP2* mRNA and LRP2 protein levels in CPTAC. Figure S3: Immunohistochemical detection of LRP2 in epithelial cells of proximal tubules in human kidney cortex. Figure S4: Immunohistochemical detection of LRP2 in ductal cells in healthy human glandular breast epithelium. Figure S5: High-magnification analyses of LRP2 expression and localization in human glandular breast epithelium sample #2. Figure S6: Immunohistochemical analysis of LRP2 expression in 12 different luminal A invasive ductal breast carcinomas. Figure S7: Low *LRP2* is associated with poor survival in additional non-TCGA cohorts. Figure S8: Correlation between *LRP2* expression and cg0263127 methylation across cancer types. Figure S9: cg02361027 is located within a highly conserved region of the *LRP2* gene. Table S1: List of TCGA cancer type abbreviations. Table S2: Source data for TCGA Pan-Cancer *LRP2* survival analysis. Table S3: Differential gene-expression analysis between *LRP2*^low^ and *LRP2*^high^ tumors in KIRC, KIRP and BRCA. Table S4: Gene Set Enrichment Analysis of differentially expressed genes between *LRP2*^low^ and *LRP2*^high^ tumors in KIRC, KIRP and BRCA. Table S5: Pairwise associations between *LRP2* expression and clinicopathological variables. Table S6: Univariate Cox proportional-hazard survival analyses for *LRP2* and clinicopathologic variables. Table S7: Multivariate Cox proportional-hazards analyses with LRP2 expression and clinicopathological variables as covariates.
