# Supplementary material for: Epigenetic Silencing of LRP2 Is Associated with Dedifferentiation and Poor Survival in Multiple Solid Tumor Types
Source: Cancers (Basel). 2023 Mar 17;15(6):1830. doi: 10.3390/cancers15061830 (PMC10046670; doi:10.3390/cancers15061830)
Supplement: Supplementary file 1 [file cancers-15-01830-s001.zip › Supplementary Tables and Figures.docx]

Supplementary Materials: Epigenetic Silencing of *LRP2* Is
Associated with Dedifferentiation and Poor Survival in
Multiple Solid Tumor Types

Martin Q. Rasmussen, Gitte Tindbæk, Morten Muhlig Nielsen, Camilla Merrild, Torben Steiniche,
Jakob Skou Pedersen, Søren K. Moestrup, Søren E. Degn and Mette Madsen

**Figure S1.** *LRP2* expression is restricted to epithelial cells in the breast tumor microenvironment. (**A**–**B**). *LRP2* expression (normalized) in single cells across major cell types from single cell RNA sequencing of human breast cancer (Wu et al., 2021) shown with violin plots (panel **A**) or dot plots (panel **B**). C-D. *LRP2* expression (normalized) in single cells across minor epithelial cell types from single cell RNA sequencing of human breast cancer (Wu et al., 2021) shown with violin plots (panel **C**) or dot plots (panel **D**). Luminal Progenitors, Myoepithelial and Mature Luminal are minor cell types of Normal Epithelial cells. Cancer Cycling, Cancer Basal SC, Cancer Her2 SC, Cancer LumB SC and Cancer LumA SC are minor cell types of Cancer Epithelial cells. Dot sizes represent the % of cells in the cluster where *LRP2* expression was detected. Dot colors represent the average expression of *LRP2* in the cluster.


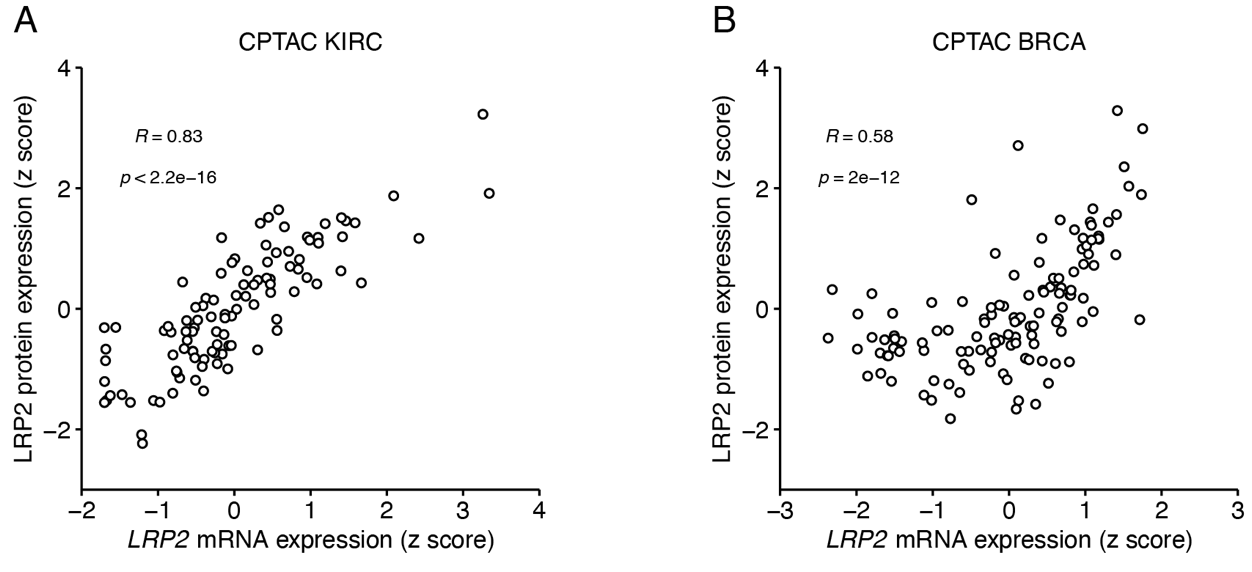


**Figure S2.** LRP2 mRNA and protein levels are strongly correlated in human cancers. (**A**). Scatter plot of *LRP2* mRNA expression (z score) and LRP2 protein expression (z score) from clear cell renal cell carcinoma tumors (*n* = 103) in CPTAC. (**B**). Scatter plot of *LRP2* mRNA expression (z score) and LRP2 protein expression (z score) from breast cancer tumors (*n* = 122) in CPTAC. Pearson’s R and corresponding P values are shown.


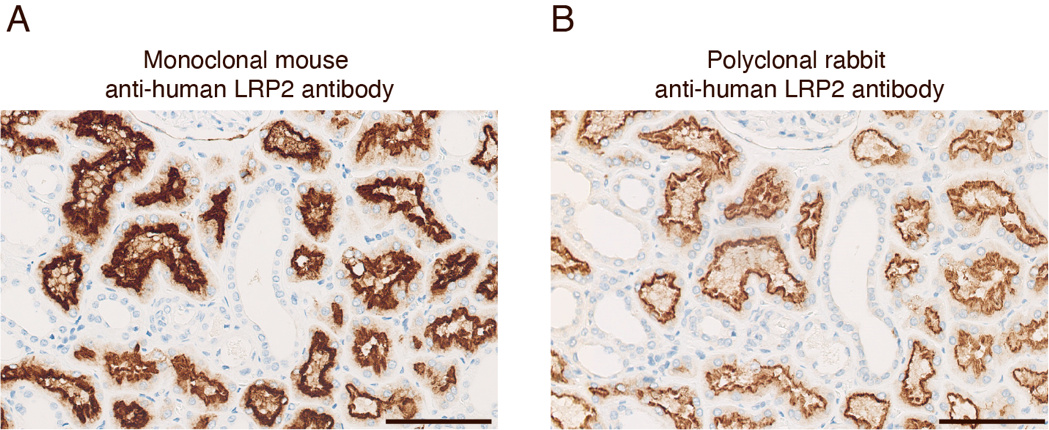


**Figure S3.** Immunohistochemical detection of LRP2 in epithelial cells of proximal tubules in human kidney cortex. (**A**). Section of formalin-fixed paraffin-embedded human kidney cortex labelled with a monoclonal mouse anti-human LRP2 antibody. (**B**). Section of formalin-fixed paraffin-embedded human kidney cortex labelled with a polyclonal rabbit anti-human LRP2 antibody. Scale bar: 100 µm.


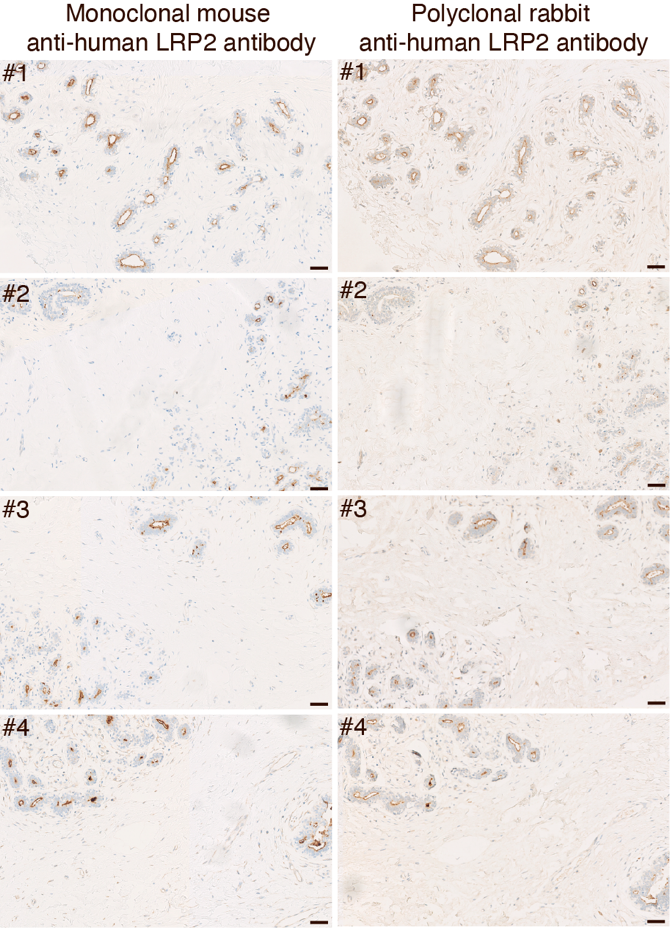


**Figure S4.** Immunohistochemical detection of LRP2 in ductal cells in healthy human glandular breast epithelium. Representative images of sections of four different formalin-fixed paraffin-embedded human glandular breast epithelium samples labelled with a monoclonal mouse anti-human LRP2 antibody (left panels) or a polyclonal rabbit anti-human LRP2 antibody (right panels). Scale bar: 50 µm.

_
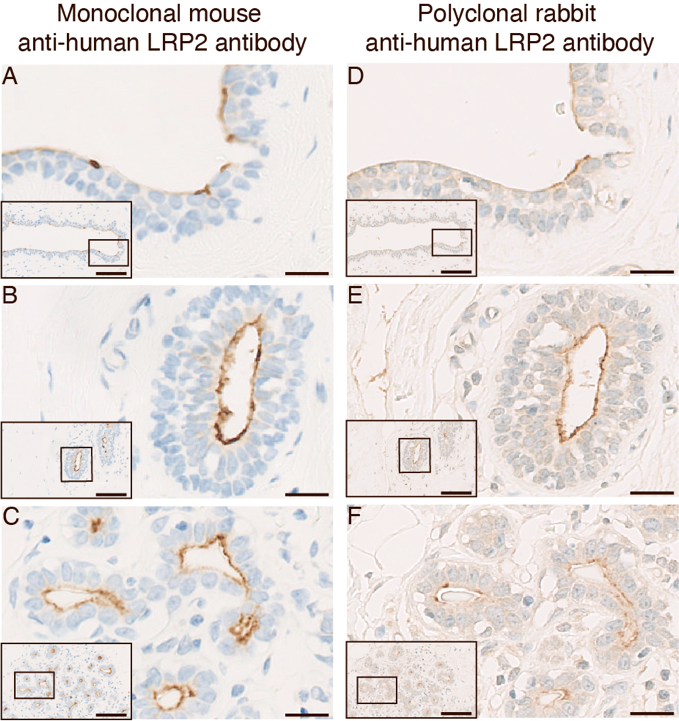
_

**Figure S5.** Zoom analyses of LRP2 expression and localization in human glandular breast epithelium sample #2. Zoomed images of sections of a formalin-fixed paraffin-embedded healthy human glandular breast epithelium sample #2 labelled with a monoclonal mouse anti-human LRP2 antibody (**A**–**C**) or a polyclonal rabbit anti-human LRP2 antibody (**D**–**F**). Scale bar in small images: 100 µm. Scale bar in large images: 20 µm. Note the predominant localization of LRP2 at the apical membrane of ductal epithelial cells.


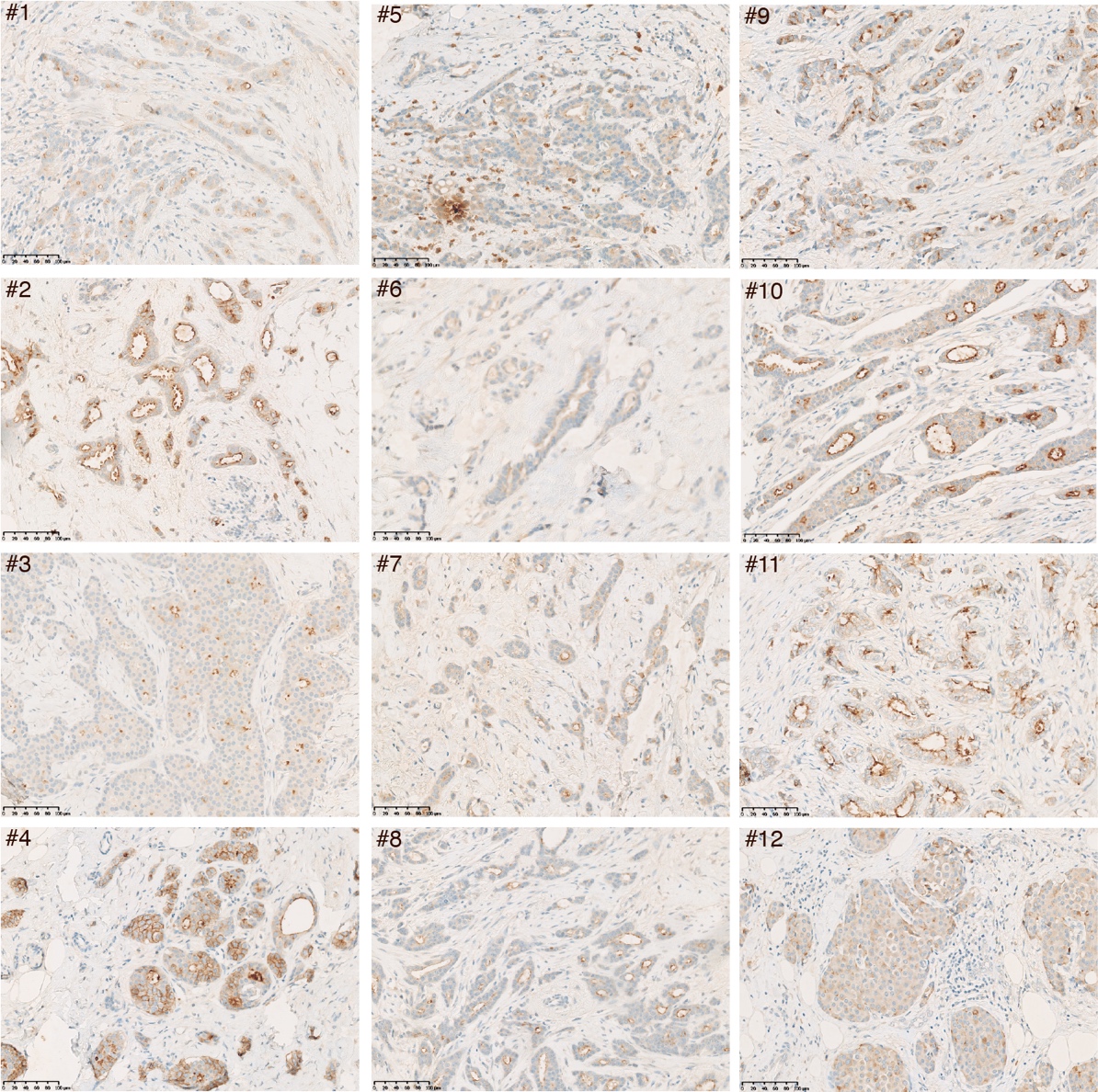


**Figure S6.** Immunohistochemical analysis of LRP2 expression in 12 different human luminal A invasive ductal breast carcinomas. Representative images of sections of 12 different formalin-fixed paraffin-embedded human luminal A invasive ductal breast carcinomas labelled with a polyclonal rabbit anti-human LRP2 antibody are shown. Scale bar:100 µm. Similar images of labelling of sections of the same 12 luminal A invasive ductal carcinomas labelled with a monoclonal mouse anti-human LRP2 antibody are shown in main Figure 3.


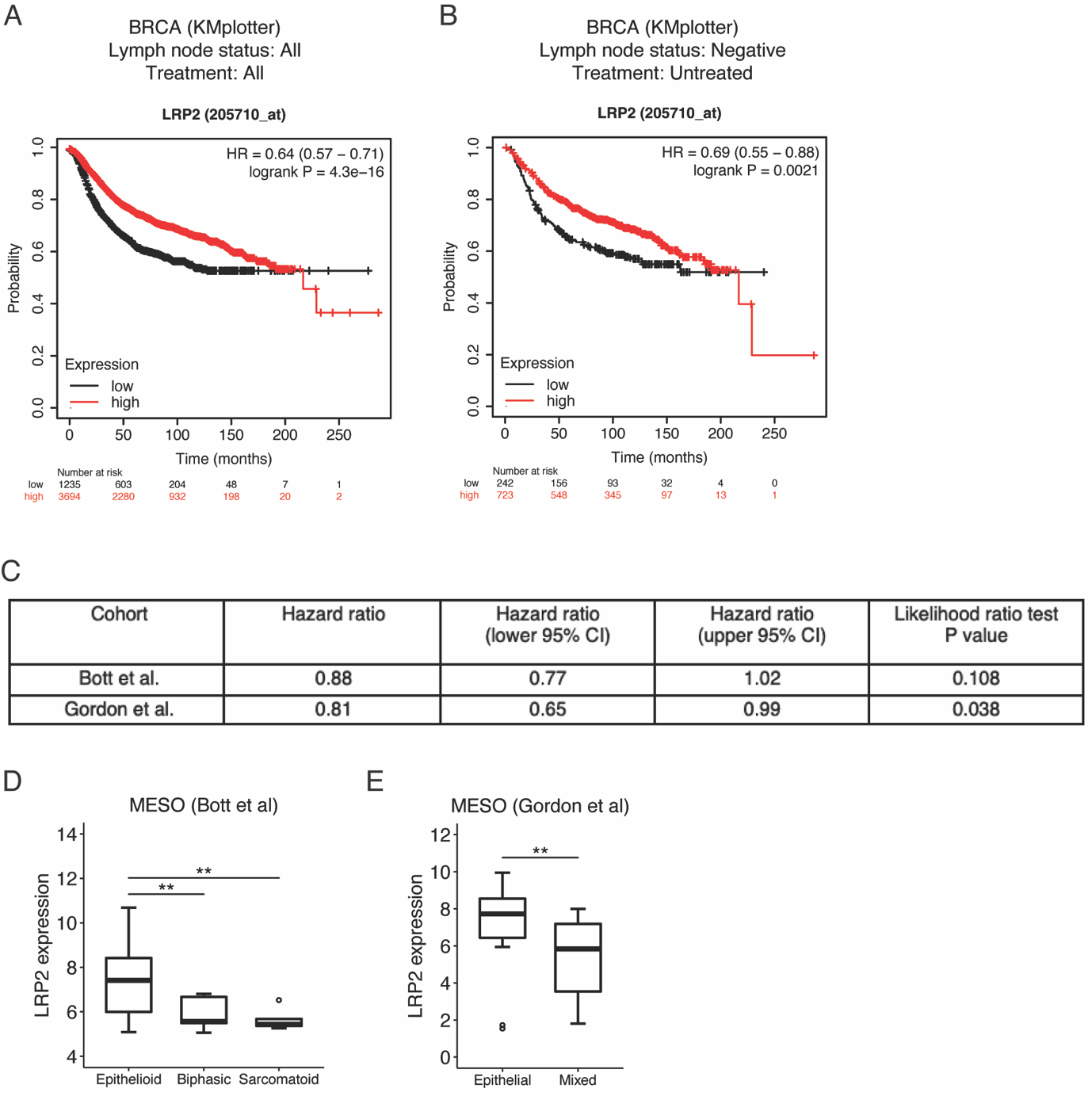


**Figure S7.** Low *LRP2* is associated with poor survival in additional non-TCGA cohorts. (**A**–**F**). Kaplan-Meier curves of *LRP2*^high^ and *LRP2*^low^ groups stratified based on a lower quartile cutoff (upper panel) and risk table (lower panel) in each dataset. (**A)**. Includes all patients in the KMplotter breast cancer microarray sequencing cohort (*n* = 4939). (**B**). Includes patients without lymph node metastasis and no prior systemic treatment in the KMplotter breast cancer microarray cohort (*n* = 965). (**C**). Table of results from univariate cox proportional hazards modeling using *LRP2* expression in the mesothelioma microarray cohorts from Bott et al. (*n* = 49) and Gordon et al. (*n* = 50). (**D**). Boxplot of *LRP2* expression across mesothelioma histological subtypes defined in Gordon et al. (**E**). Boxplot of *LRP2* expression across mesothelioma histological subtypes defined in Bott et al. Boxplot lines represent lower quartile, median and upper quartile. Whiskers represent 1.5 times above or below interquartile range. Points reflect outliers. Wilcox rank sum test: * *P* ≤ 0.05, ** *P* ≤ 0.01, *** *P* ≤ 0.001, **** *P* ≤ 0.0001.


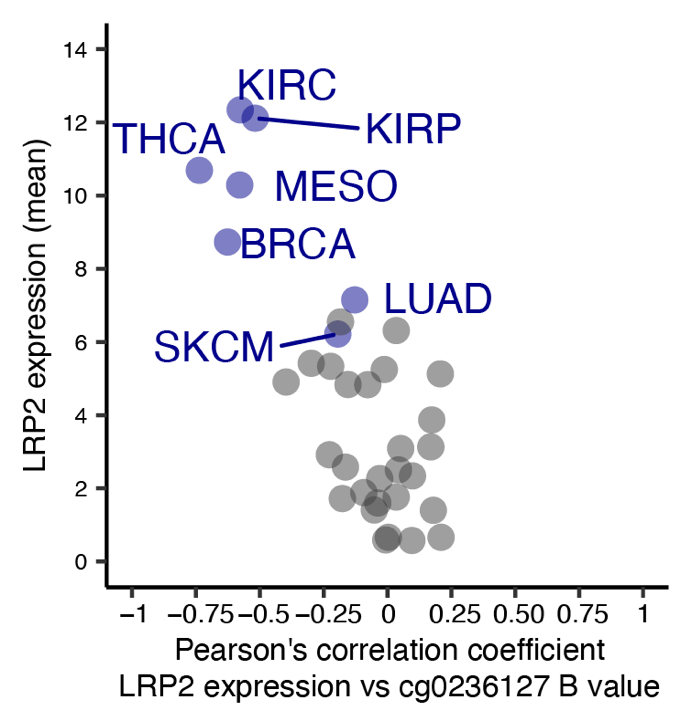


**Figure S8.** Correlation between *LRP2* expression and cg0263127 methylation across cancer types. Scatter plot of the Pearson’s correlation coefficient between LRP2 expression and cg0236127 (x axis) and mean *LRP2* expression (y axis) for each cancer type in the TCGA PAN-CANCER dataset. Cancer types where low *LRP2* is associated with poor survival are labeled in blue.


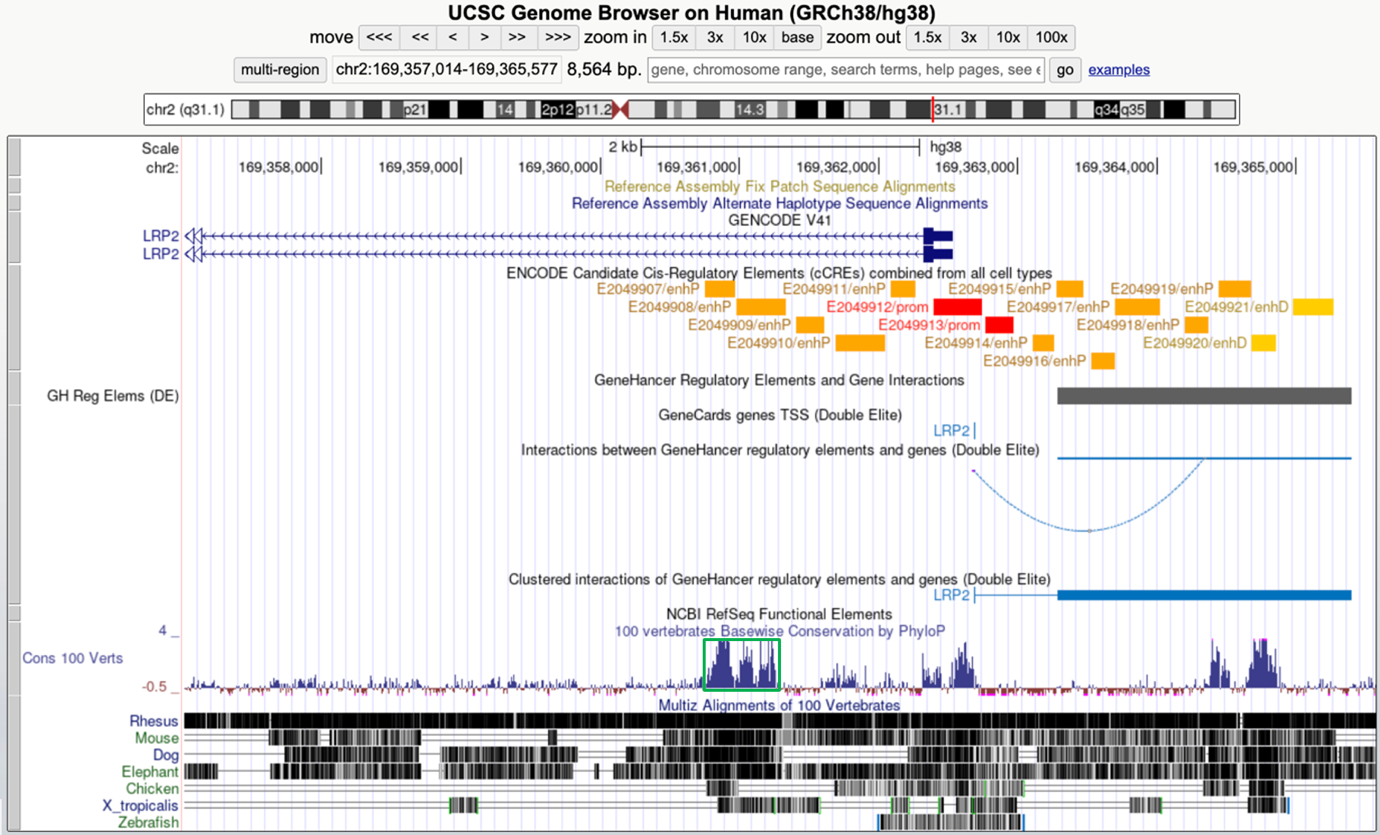


**Figure S9.** cg02310627 is located in a highly conserved region of the first intron in the *LRP2* gene. Screenshot from UCSC Genome Browser (GRCh38) for chromosome 2 positions 169,357,014-169,365,577 containing the *LRP2* gene. cg0231627 is located on position 169360890 within a highly conserved region, as indicated in the Cons 100 Verts track with a green box.

**Table S1.** List of TCGA (The Cancer Genome Atlas) cancer type abbreviations.

| LAML | Acute Myeloid Leukemia |
| --- | --- |
| ACC | Adrenocortical carcinoma |
| BLCA | Bladder Urothelial Carcinoma |
| LGG | Brain Lower Grade Glioma |
| BRCA | Breast invasive carcinoma |
| CESC | Cervical squamous cell carcinoma and endocervical adenocarcinoma |
| CHOL | Cholangiocarcinoma |
| LCML | Chronic Myelogenous Leukemia |
| COAD | Colon adenocarcinoma |
| CNTL | Controls |
| ESCA | Esophageal carcinoma |
| GBM | Glioblastoma multiforme |
| HNSC | Head and Neck squamous cell carcinoma |
| KICH | Kidney Chromophobe |
| KIRC | Kidney renal clear cell carcinoma |
| KIRP | Kidney renal papillary cell carcinoma |
| LIHC | Liver hepatocellular carcinoma |
| LUAD | Lung adenocarcinoma |
| LUSC | Lung squamous cell carcinoma |
| DLBC | Lymphoid Neoplasm Diffuse Large B-cell Lymphoma |
| MESO | Mesothelioma |
| MISC | Miscellaneous |
| OV | Ovarian serous cystadenocarcinoma |
| PAAD | Pancreatic adenocarcinoma |
| PCPG | Pheochromocytoma and Paraganglioma |
| PRAD | Prostate adenocarcinoma |
| READ | Rectum adenocarcinoma |
| SARC | Sarcoma |
| SKCM | Skin Cutaneous Melanoma |
| STAD | Stomach adenocarcinoma |
| TGCT | Testicular Germ Cell Tumors |
| THYM | Thymoma |
| THCA | Thyroid carcinoma |
| UCS | Uterine Carcinosarcoma |
| UCEC | Uterine Corpus Endometrial Carcinoma |
| UVM | Uveal Melanoma |

**Table S2.** Source data for TCGA Pan-Cancer LRP2 survival analysis.

| **Cancer Type** | **Survival Parameter** | **Hazard Ratio** | **Hazard Ratio  95 % CI (low)** | **Hazard Ratio  95 % CI (high)** | **Wald  Statistic** | **Likelihood Ratio  Test *P* Value** |
| --- | --- | --- | --- | --- | --- | --- |
| ACC | OS | 1,387986 | 1,027407 | 1,875115 | 0,032669 | 0,037525 |
| BLCA | OS | 0,991956 | 0,925103 | 1,063640 | 0,820514 | 0,820221 |
| CESC | OS | 0,890101 | 0,784015 | 1,010541 | 0,072175 | 0,055707 |
| CHOL |  |  |  |  |  |  |
| COAD | OS | 1,210005 | 1,054675 | 1,388211 | 0,006541 | 0,010757 |
| DLBC |  |  |  |  |  |  |
| ESCA | OS | 1,070379 | 0,976167 | 1,173682 | 0,147943 | 0,159030 |
| GBM | OS | 1,011164 | 0,935109 | 1,093403 | 0,780803 | 0,780793 |
| HNSC | OS | 1,088713 | 1,004384 | 1,180123 | 0,038800 | 0,043074 |
| KICH |  |  |  |  |  |  |
| KIRC | OS | 0,914282 | 0,875866 | 0,954382 | 0,000043 | 0,000177 |
| KIRP | OS | 0,883923 | 0,812594 | 0,961514 | 0,004051 | 0,009661 |
| LAML | OS | 1,061628 | 0,964716 | 1,168276 | 0,220771 | 0,222868 |
| LGG | PFI | 1,049606 | 0,982789 | 1,120966 | 0,149122 | 0,145539 |
| LIHC | OS | 1,063452 | 1,005824 | 1,124383 | 0,030445 | 0,032892 |
| LUAD | OS | 0,947129 | 0,898452 | 0,998443 | 0,043609 | 0,045570 |
| LUSC | OS | 1,058081 | 0,996340 | 1,123649 | 0,065706 | 0,066196 |
| MESO | OS | 0,869444 | 0,799038 | 0,946052 | 0,001166 | 0,001860 |
| METABRIC | OS | 0,898236 | 0,851270 | 0,947792 | 0,000090 | 0,000065 |
| OV | OS | 1,000749 | 0,960820 | 1,042336 | 0,971268 | 0,971268 |
| PAAD | OS | 0,988032 | 0,879124 | 1,110431 | 0,839864 | 0,839250 |
| PCPG |  |  |  |  |  |  |
| PRAD |  |  |  |  |  |  |
| READ |  |  |  |  |  |  |
| SARC | OS | 1,112505 | 0,995849 | 1,242826 | 0,059246 | 0,067076 |
| SKCM | PAS | 0,952537 | 0,917046 | 0,989400 | 0,012072 | 0,011890 |
| STAD | OS | 1,044919 | 0,986726 | 1,106545 | 0,132864 | 0,140322 |
| TGCT | PFI | 1,103232 | 0,981924 | 1,239528 | 0,098324 | 0,099646 |
| THCA | DFI | 0,815125 | 0,680590 | 0,976254 | 0,026345 | 0,041057 |
| THYM |  |  |  |  |  |  |
| UCEC | OS | 1,045838 | 0,939278 | 1,164487 | 0,413682 | 0,416023 |
| UCS | OS | 1,066044 | 0,935806 | 1,214407 | 0,336056 | 0,333593 |
| UVM | OS | 1,030394 | 0,885401 | 1,199131 | 0,698790 | 0,698541 |

Statistical output from the univariate cox proportional hazards model are shown. Cancer types with a likelihood ratio test *P* value < 0.05 and hazard ratio > 1 are highlighted in red. Cancer types with likelihood ratio test *P* value < 0.05 and hazard ratio < 1 are highlighted in blue. *P* values are uncorrected. No data is shown for cancer types with insufficient survival data for analysis as determined by The Cancer Genome Atlas working group.

**Table S5.** Pairwise associations between LRP2 expression and clinicopathological variables.

| KIRC | | | | | | | | |
| --- | --- | --- | --- | --- | --- | --- | --- | --- |
|  | Age | Gender:  Female | Gender: Male | Stage:  Low (I or II) | | Stage:  High (III or IV) | Fuhrman grade: Low (G1 or G2) | Fuhrman grade: High (G3 or G4) |
| LRP2: Low | 61,67 | 32 | 93 | 63 | | 62 | 49 | 76 |
| LRP2: High | 60,36 | 150 | 245 | 254 | | 141 | 191 | 204 |
| P value | 0,29 | 0,02 | | 0,01 | | | 0,09 | |
| KIRP | | | | | | | | |
|  | Age | Gender:  Female | Gender: Male | Stage:  Low (I or II) | | Stage:  High (III or IV) |  |  |
| LRP2: Low | 58,48 | 29 | 32 | 41 | | 20 |  |  |
| LRP2: High | 62,49 | 39 | 159 | 151 | | 47 |  |  |
| P value | 0,03 | 3,26E-05 | | 0,21 | | |  |  |
| MESO | | | | | | | | |
|  | Age | Gender: Female | Gender: Male | Stage:  Low (I or II) | | Stage:  High (III or IV) |  |  |
| LRP2: Low | 64,91 | 4 | 18 | 8 | | 14 |  |  |
| LRP2: High | 62,34 | 12 | 53 | 18 | | 47 |  |  |
| P value | 0,24 | 1,00 | | 0,62 | | |  |  |
| THCA | | | | | | | | |
|  | Age | Gender: Female | Gender: Male | Stage:  Low (I or II) | | Stage:  High (III or IV) |  |  |
| LRP2: Low | 48,23 | 90 | 38 | 73 | | 55 |  |  |
| LRP2: High | 46,89 | 282 | 102 | 267 | | 115 |  |  |
| P value | 0,40 | 0,57 | | 0,01 | | |  |  |
| METABRIC | | | | | | | | |
|  | Age | NPI:  Good  (<3.4) | NPI:  Moderate to poor (>3.4) | |  |  |  |  |
| LRP2: Low | 60,28 | 83 | 393 | |  |  |  |  |
| LRP2: High | 61,35 | 557 | 871 | |  |  |  |  |
| P value | 0,13 | 1,03E-17 | | |  |  |  |  |
| METABRIC (ER+) | | | | | | | | |
|  | Age | NPI:  Good (<3.4) | NPI:  Moderate to poor (>3.4) | |  |  |  |  |
| LRP2: Low | 64,05 | 56 | 196 | |  |  |  |  |
| LRP2: High | 63,10 | 527 | 666 | |  |  |  |  |
| *P* value | 0,26 | 1,73E-10 | | |  |  |  |  |

Pairwise associations between LRP2 expression and clinicopathological variables in KIRC, KIRP, MESO, THCA, METABRIC and ER+ METABRIC. LRP2 expression was dichotomized by the lower quartile. Student’s *t*-test was used to test the association between LRP2 expression groups and age. χ^2^ test was used to test the association between LRP2 expression groups and gender, AJCC pathological stage, Fuhrman grade (for KIRP), and Nottingham Prognostic Index (NPI) (for METABRIC).

**Table S6.** Univariate Cox proportional hazard survival analyses for LRP2 and clinicopathologic variables.

| KIRC | | | | | |
| --- | --- | --- | --- | --- | --- |
|  | Hazard Ratio | Hazard Ratio  (95% CI lower) | Hazard Ratio  (95% CI upper) | Wald Statistic | Likelihood ratio test *P* value |
| LRP2: High | 0,55 | 0,40 | 0,75 | 1,66E-04 | 2,84E-04 |
| Age: Increasing | 1,03 | 1,02 | 1,04 | 1,15E-05 | 1,05E-05 |
| Gender: Male | 0,96 | 0,70 | 1,31 | 7,90E-01 | 7,90E-01 |
| Stage: High (III or IV) | 3,70 | 2,69 | 5,08 | 6,24E-16 | 5,10E-17 |
| Fuhrman grade:  High (G3 or G4) | 2,55 | 1,82 | 3,59 | 6,93E-08 | 1,04E-08 |
| KIRP | | | | | |
|  | Hazard Ratio | Hazard Ratio  (95% CI lower) | Hazard Ratio  (95% CI upper) | Wald Statistic | Likelihood Ratio Test *P* Value |
| LRP2: High | 0,36 | 0,19 | 0,68 | 1,83E-03 | 3,17E-03 |
| Age: Increasing | 1,00 | 0,97 | 1,03 | 9,27E-01 | 9,27E-01 |
| Gender: Male | 0,61 | 0,30 | 1,21 | 1,54E-01 | 1,68E-01 |
| Stage: High (III or IV) | 6,23 | 3,23 | 11,98 | 4,45E-08 | 2,42E-08 |
| MESO | | | | | |
|  | Hazard Ratio | Hazard Ratio  (95% CI lower) | Hazard Ratio  (95% CI upper) | Wald Statistic | Likelihood Ratio Test *P* Value |
| LRP2: High | 0,51 | 0,30 | 0,86 | 1,18E-02 | 1,66E-02 |
| Age: Increasing | 1,02 | 0,99 | 1,05 | 1,66E-01 | 1,61E-01 |
| Gender: Male | 0,89 | 0,49 | 1,60 | 6,91E-01 | 6,94E-01 |
| Stage: High (III or IV) | 0,99 | 0,59 | 1,68 | 9,78E-01 | 9,78E-01 |
| THCA | | | | | |
|  | Hazard Ratio | Hazard Ratio  (95% CI lower) | Hazard Ratio  (95% CI upper) | Wald Statistic | Likelihood Ratio Test *P* Value |
| LRP2: High | 0,45 | 0,21 | 0,98 | 4,43E-02 | 5,22E-02 |
| Age: Increasing | 0,99 | 0,97 | 1,02 | 6,49E-01 | 6,46E-01 |
| Gender: Male | 1,23 | 0,52 | 2,91 | 6,40E-01 | 6,46E-01 |
| Stage: High (III or IV) | 1,59 | 0,69 | 3,63 | 2,73E-01 | 2,89E-01 |
| METABRIC | | | | | |
|  | Hazard Ratio | Hazard Ratio  (95% CI lower) | Hazard Ratio  (95% CI upper) | Wald Statistic | Likelihood Ratio Test *P* Value |
| LRP2: High | 0,71 | 0,62 | 0,81 | 3,21E-07 | 6,61E-07 |
| Age: Increasing | 1,04 | 1,03 | 1,04 | 5,22E-40 | 4,92E-42 |
| NPI: Moderate to poor (>3.4) | 1,59 | 1,40 | 1,81 | 2,35E-12 | 5,52E-13 |
| METABRIC (ER+) | | | | | |
|  | Hazard Ratio | Hazard Ratio  (95% CI lower) | Hazard Ratio  (95% CI upper) | Wald Statistic | Likelihood Ratio Test *P* Value |
| LRP2: High | 0,69 | 0,58 | 0,82 | 1,60E-05 | 3,30E-05 |
| Age: Increasing | 1,05 | 1,04 | 1,06 | 1,42E-51 | 9,73E-56 |
| NPI: Moderate to poor (>3.4) | 1,70 | 1,48 | 1,96 | 2,04E-13 | 6,01E-14 |

Univariate Cox proportional hazard survival analyses for LRP2 and clinicopathologic variables in KIRC, KIRP, MESO, THCA, METABRIC and ER+ METABRIC. LRP2 expression was dichotomized by the lower quartile. Hazard ratios and 95% confidence intervals (CI) are shown together with Wald statistics and Likelihood ratio test *P* values.

**Table S7.** Multivariate Cox proportional hazards analyses with LRP2 expression and clinicopathological variables as covariates.

| KIRC | | | | |
| --- | --- | --- | --- | --- |
|  | Hazard Ratio | Hazard Ratio  (95% CI lower) | Hazard Ratio  (95% CI upper) | *P* Value |
| LRP2: High | 0,64 | 0,46 | 0,87 | 5,28E-03 |
| Age: Increasing | 1,03 | 1,01 | 1,04 | 6,58E-05 |
| Stage: High (III or IV) | 3,04 | 2,18 | 4,25 | 6,27E-11 |
| Fuhrman grade: High (III or IV) | 1,67 | 1,17 | 2,40 | 4,96E-03 |
| KIRP | | | | |
|  | Hazard Ratio | Hazard Ratio  (95% CI lower) | Hazard Ratio  (95% CI upper) | *P* Value |
| LRP2: High | 0,25 | 0,13 | 0,50 | 8,90E-05 |
| Age: Increasing | 1,01 | 0,98 | 1,04 | 4,76E-01 |
| Stage: High (III or IV) | 7,50 | 3,84 | 14,63 | 3,46E-09 |
| MESO | | | | |
|  | Hazard Ratio | Hazard Ratio  (95% CI lower) | Hazard Ratio  (95% CI upper) | *P* Value |
| LRP2: High | 0,52 | 0,31 | 0,88 | 1,47E-02 |
| Age: Increasing | 1,02 | 0,99 | 1,05 | 2,07E-01 |
| Stage: High (III or IV) | 0,99 | 0,59 | 1,68 | 9,71E-01 |
| THCA | | | | |
|  | Hazard Ratio | Hazard Ratio  (95% CI lower) | Hazard Ratio  (95% CI upper) | *P* Value |
| LRP2: High | 0,48 | 0,22 | 1,04 | 6,27E-02 |
| Age: Increasing | 0,98 | 0,95 | 1,01 | 2,87E-01 |
| Stage: High (III or IV) | 2,05 | 0,73 | 5,80 | 1,75E-01 |
| METABRIC | | | | |
|  | Hazard Ratio | Hazard Ratio  (95% CI lower) | Hazard Ratio  (95% CI upper) | *P* Value |
| LRP2: High | 0,76 | 0,66 | 0,87 | 6,94E-05 |
| Age: Increasing | 1,04 | 1,03 | 1,04 | 1,22E-42 |
| NPI: Moderate to poor (>3.4) | 1,60 | 1,40 | 1,82 | 3,94E-12 |
| METABRIC (ER+) | | | | |
|  | Hazard Ratio | Hazard Ratio  (95% CI lower) | Hazard Ratio  (95% CI upper) | *P* Value |
| LRP2: High | 0,80 | 0,68 | 0,95 | 1,26E-02 |
| Age: Increasing | 1,05 | 1,04 | 1,06 | 2,17E-48 |
| NPI: Moderate to poor (>3.4) | 1,56 | 1,35 | 1,80 | 1,54E-09 |

Multivariate Cox proportional hazards analyses with LRP2 expression and clinicopathological variables as covariates in KIRC, KIRP, MESO, THCA, METABRIC and ER+ METABRIC. LRP2 was dichotomized by the lower quartile. Hazard ratios and 95% confidence intervals (CI) and P values are shown for each variable.

**Table S3** (separate Excel file “Supplementary Table S3.xlsx”).

**Table S4** (separate Excel file “Supplementary Table S4.xlsx”).
